# The potential of facultative predatory *Actinomycetota* spp. and prospects in agricultural sustainability

**DOI:** 10.3389/fmicb.2022.1081815

**Published:** 2023-01-25

**Authors:** Manar Ibrahimi, Souad Loqman, Martin Jemo, Mohamed Hafidi, Laurent Lemee, Yedir Ouhdouch

**Affiliations:** ^1^Laboratory of Molecular Chemistry, Materials and Catalysis, Faculty of Sciences and Technics, Sultan Moulay Slimane University, Beni-Mellal, Morocco; ^2^Higher School of Technology Fkih Ben Salah, Sultan Moulay Slimane University, Fkih Ben Salah, Morocco; ^3^Laboratory of Microbiology and Virology, Faculty of Medicine and Pharmacy, Cadi Ayyad University, Marrakesh, Morocco; ^4^AgroBiosciences Program, Mohammed VI Polytechnic University (UM6P), Ben Guerir, Morocco; ^5^Labelled Research Unit N°4 CNRST, Laboratory of Microbial Biotechnologies, Agrosciences and Environment (BioMAgE), Faculty of Sciences Semlalia, Cadi Ayyad University, Marrakesh, Morocco; ^6^Institut de Chimie des Milieux et Matériaux de Poitiers (IC2MP–CNRS UMR 7285), Université de Poitiers, Poitiers, France

**Keywords:** bacteria, interactions, diversity, ecology, survival mechanism, prokaryotic predation

## Abstract

*Actinomycetota* in the phylum of bacteria has been explored extensively as a source of antibiotics and secondary metabolites. In addition to acting as plant growth-promoting agents, they also possess the potential to control various plant pathogens; however, there are limited studies that report the facultative predatory ability of *Actinomycetota* spp. Furthermore, the mechanisms that underline predation are poorly understood. We assessed the diversity of strategies employed by predatory bacteria to attack and subsequently induce the cell lysing of their prey. We revisited the diversity and abundance of secondary metabolite molecules linked to the different predation strategies by bacteria species. We analyzed the pros and cons of the distinctive predation mechanisms and explored their potential for the development of new biocontrol agents. The facultative predatory behaviors diverge from group attack “wolfpack,” cell-to-cell proximity “epibiotic,” periplasmic penetration, and endobiotic invasion to degrade host-cellular content. The epibiotic represents the dominant facultative mode of predation, irrespective of the habitat origins. The wolfpack is the second-used approach among the *Actinomycetota* harboring predatory traits. The secondary molecules as chemical weapons engaged in the respective attacks were reviewed. We finally explored the use of predatory *Actinomycetota* as a new cost-effective and sustainable biocontrol agent against plant pathogens.

## 1. Introduction

Cellular tropism also referred to as “cellular predation,” is a regular interspecific antagonistic that occurs in diverse living habitats. It also defines an act of a predatory organism to kill and devour a prey organism for its nutritional requirements (Pérez et al., 2016). Predation behavior expands from primitive prokaryotic microbes to highly evolved mammals in the animal kingdom (Sinclair et al., 2003; Ripple and Beschta, 2004). Among the microorganisms, a family member of the virus, bacteria have developed predatory behaviors that are well investigated under *in vivo* and *ex vivo* conditions (Curds, 1982; Gonzalez et al., 1990; Parry, 2004). Myxobacteria and *Bdellovibrio* are δ*-Proteobacteria* in the *bacteria* phyla with well-known bacteriophagic nature (Stolp and Starr, 1963; Casida, 1982; Berleman et al., 2006; Martins et al., 2022). Through predation attitude, *Myxobacteria*, and *Bdellovibrio* contribute to community structuring and carbon recycling in the soil food web systems and play an important ecosystem function are well-known keystone taxa (Bratanis et al., 2020; Cavallo et al., 2021; Mookherjee and Jurkevitch, 2022; Ogundero et al., 2022; Whitworth, 2022; Wu et al., 2022). Thus, bacteria species in the actinomycetes species are gram-positive and mostly studied for secondary metabolites production and were recently discovered to exert a facultative predatory role (Zeph and Casida, 1986; Hoshino et al., 2015; Ait Barka et al., 2016; Katz and Baltz, 2016; Baltz, 2019; Ouchari et al., 2019; Ibrahimi et al., 2020; Korichi et al., 2021). Studies deciphering or investigating the facultative predation role of *Actinomycetota* are gaining growing research attention (Kumbhar et al., 2014; Ibrahimi et al., 2019, 2020; Baig et al., 2021) owing to their lifestyles adjustment to adapt to complex limited resources (Ibrahimi et al., 2019, 2020) and importantly potential application to design bio-pesticides molecules/products alternatives to heavy toxic pesticides inorganic molecules (Palaniyandi et al., 2013). The approach is driven by increasing consumer demands for safe, healthy, and organically produced foods globally (Alvarez et al., 2017, Sathya et al., 2017; AbdElgawad et al., 2020). However, in the context of *Actinomycetota* species, the mechanisms that underline opportunistic p*redation* behaviors *are under*-investigated (Ibrahimi et al., 2019, 2020).

Predation is a bacteria co-evolutional trait in the *Actinomycetota*, owing to adjustments in lifestyles among the species, possible geographical local adaptations, and habitat change (Jousset, 2012; Ibrahimi et al., 2020). For a long time, *Actinomycetota* were only viewed as competitive, rather than predatory organisms (Kumbhar and Watve, 2013; Kumbhar et al., 2014; Ibrahimi et al., 2020; Pérez et al., 2020). As a consequence, despite their widespread ecological importance in the environment, there are very few investigations on *Actinomycetota* predation (Bentley et al., 2002; Mawang et al., 2021; Boubekri et al., 2022; Santos-Aberturas and Vior, 2022). The first evidence of the *Actinomycetota* opportunistic predation behaviors is described in the *Streptomyces* and *Agromyces* genera (Waksman and Woodruff, 1941; Casida, 1980). The author examined the utilization of *Micrococcus luteus* as prey by *Streptomyces* species. As versatile-opportunistic *Actinomycetota*, *Streptomyces* is also a non-obligate epibiotic predator of various microorganisms, specifically, *Staphylococcus aureus*, *Escherichia coli*, *Bacillus* sp., *Pseudomonas aeruginosa*, and *Klebsiella* sp. (Kumbhar et al., 2014). In addition, It was reported in the literature that under *in vivo* conditions, *Streptomyces* isolates exhibited the predatory ability on various prey bacteria cells (Gram+, Gram−) and multidrug-resistant strains (Ibrahimi et al., 2020). The latest supportive evidence of good predatory behavior by *Streptomyces* against various types of prey is reported by Baig et al. (2021). However, studies examining the mechanisms that underline predator–prey relationships and the diversity or identity of small signal molecules are lacking (Ibrahimi et al., 2020).

Increasing plant diseases due to pathogenic microbes represent an important global constraint for agricultural production and economic losses (Collinge and Sarrocco, 2022). Current interventions are toward the use of synthetic heavy toxic molecules pesticides for crop protection that negatively poses acute risks to human health and the environment (Rani et al., 2021). It is imperative to find alternative solutions for a sustainable crop yield (Collinge and Sarrocco, 2022). The increasing knowledge and understanding of plant–microbe interactions, in particular the predatory capability of *Actinomycetota* to design as a biopesticide product to combat plant-associated pathogenic microbes (Shivlata and Satyanarayana, 2017). The objective of the present study was to:

a) critically review the facultative predatory mechanisms of bacteria and *Actinomycetota*;
b) discuss the various small molecules synthesized from bacteria and *Actinomycetota* species during their opportunistic predatory lifestyle stage; and
c) explore the potential beneficial use of *Actinomycetota* synthesized small molecules during the predation stages in the development of biocontrol agents for plant disease suppression and protection.

## 2. Mechanisms of predation by bacteria and *Actinomycetota* species

**FIGURE 1:**
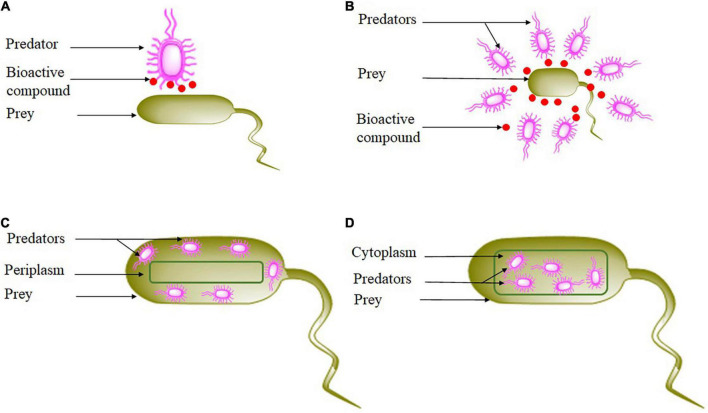
Bacterial hunting strategies. **(A)** Epibiotic strategy, **(B)** wolfpack strategy, **(C)** periplasmic strategy, and **(D)** endobiotic strategy.

Predation by bacteria has traditionally attracted lower attention than their multicellular development or their production of bioactive compounds (Furness et al., 2020). In recent years, many aspects of bacterial predation are starting to be explored through research (Table 1). Since the purpose of a predatory bacteria is to kill and digest its prey, it remains necessary to understand the hunting and killing behavior of the predator. Most authors propose to classify bacterial hunting strategies into four general categories: epibiotic, group attack, or wolfpack, periplasmic penetration, and endobiotic predation or direct invasion (Martin, 2002; Jurkevitch, 2007; Berleman and Kirby, 2009; Pasternak et al., 2014; Pérez et al., 2016). Epibiotic is a tactic that requires close cell-to-cell proximity (Shi et al., 1993). When the predation is extracellular, the predator attached to the prey from outside does not invade either the periplasm or the cytoplasm of the prey, degrades and assimilates host molecules through specialized structures, but without penetrating the prey (Martin, 2002; Pérez et al., 2016), consuming it from the exterior before dividing into the daughter cells *via* binary division (Koval et al., 2013; Figure 1). *Ensifer adhaerens* (Druga et al., 2011), *Myxococcus xanthus* (Nair, 2016; Thiery and Kaimer, 2020), and *Streptomyces* (Casida, 1988; Ibrahimi et al., 2020) are some examples of epibiotic strategy. The cell-to-cell contact between the epibiotic predator and its prey is crucial for the transfer of compounds between their cells (Castelle and Banfield, 2018; Yakimov et al., 2022). As a consequence of the limited literature on culture-based investigations, our current state of understanding of the behavior of epibiotic lifestyles is relatively poor (Bor et al., 2020; Batinovic et al., 2021; Yakimov et al., 2022). However, Cross et al. (2019) suggested that to clearly demonstrate cell-to-cell contact, conditions that could enhance predator abundance to the degree that allows detailed microscopic characterization must be evaluated.

**TABLE 1:** Predatory bacteria and their biological activity.

Group	Known habitat	Prey Gram+ Gram−		Predatory strategy	Antibiotic production	Predation type	References
**α-Proteobacteria**							
*Ensifer adhaerens*	Soil	+		Epibiotic	Yes	Facultative	Casida, 1982; Germida and Casida, 1983; Druga et al., 2011
*Micavibrio* sp.	Soil		+	Epibiotic	No	Obligate	Davidov et al., 2006; Kadouri et al., 2007
**β-Proteobacteria**							
*Cupriavidus necator*	Soil	+	+	Epibiotic	Yes	Facultative	Makkar and Casida, 1987; Kreutzer et al., 2012
*Aristabacter necator*	Soil	+	+	Epibiotic	Yes	Facultative	Casida, 1992; Cain et al., 2003
**γ-Proteobacteria**							
*Lysobacter* sp.	Soil	+	+	Wolfpack	Yes	Facultative	Li et al., 2008; Seccareccia et al., 2015
*Stenotrophomonas maltophilia*	Soil	+	+	Epibiotic	Yes	Facultative	Ryan et al., 2009; Kumbhar and Watve, 2013
*Pseudomonas*	Soil	+	+	Epibiotic	Yes	Facultative	Casida, 1992
**δ-Proteobacteria**							
*Bdellovibrionales* (d-BALOs) *Bdellovibrionaceae* *Bdellovibrio spp*.	Soil, freshwater, sewage, marine sediments perialpine lakes, marine waters, river, estuaries, terrestrial plants, gut of animals and humans		+	Periplasmic Epibiotic	Not characterized	Obligate	Cotter and Thomashow, 1992; Fratamico and Cooke, 1996; Jurkevitch and Ramati, 2000; Jurkevitch et al., 2000; Schwudke et al., 2001; Davidov et al., 2006; Jurkevitch, 2007; Sockett, 2009; Hobley et al., 2012; Ezzedine et al., 2020
*Bacteriovoracaceae* *Bacteriovorax* sp. *Peredibacter* sp.				Periplasmic	No		
Myxobacteria	Cell contact, Soil, dung, bark, Sediments	+	+	Wolfpack	Yes	Facultative	Reichenbach and Höfle, 1993; McBride and Zusman, 1996
**Chloroflexi**							
*Herpetosiphon* sp.	Cell contact freshwater	+	+	Wolfpack	Yes	Facultative	Quinn and Skerman, 1980; Trick and Lingens, 1984; Nett et al., 2006; Kiss et al., 2011
**Bacteroidetes**							
*Saprospira* sp.	Coastal sediment Sea water	+	+	Wolfpack Epibiotic	Yes	Facultative	Furusawa et al., 2003; Saw et al., 2012
*Flavobacterium*	Marine Fresh Water	+	+		Yes	Facultative	McBride et al., 2009; Banning, 2010
***Actinomycetota***							
*Streptomyces*	Soil, marine water, marine sponge Marine water	+	+	Epibiotic Wolfpack Epibiotic	Yes	Facultative	Waksman and Woodruff, 1941; Casida, 1988; Kumbhar et al., 2014; Ibrahimi et al., 2020; Baig et al., 2021
*Agromyces ramosus*	Soil		+		Yes	Facultative	Casida, 1983; Arcamone et al., 2000; Chernyakovskaya et al., 2004
*Brevibacterium*, *Glutamicibacter*, *Micromonospora*, *Nocardiopsis*, *Rhodococcus* *Rothia*	Marine sponge	+		Epibiotic	Yes	Facultative	Baig et al., 2021
**Bacillales**							
*Bacillus*	Soil		+	Epibiotic	Yes	Facultative	Gumbo et al., 2010
*Paenibacillus*	Soil Water Rhizosphere Veg. matter		+	Epibiotic	Yes	Facultative	Raza et al., 2008; Be’Er et al., 2009

The second strategy is wolfpack or group attack or group predation, predatory bacteria in this kind of predation work as a group (Marshall and Whitworth, 2019), they are assumed to hunt collectively to attack prey (Figure 1). They assemble and collectively secrete a diversity of diffusible compounds like hydrolytic enzymes and extracellular antibiotics that degrade and kill nearby bacteria. Wolfpack strategy lyse prey cells from the exterior through concerted action (Martin, 2002). Furthermore, the aim of the process of lysis is to produce small molecules that are easily assimilated by the predator (Xiao et al., 2011). Moreover, higher predatory cell densities suggest higher diffusible compounds (Keane and Berleman, 2016). The most important property of wolfpack is the lysed prey will be consumed by the predatory bacteria secreting and non-secreting (Mendes-Soares and Velicer, 2013). *Lysobacter* sp. (Hashizume et al., 2004; Li et al., 2008), *Myxobacteria* (McBride and Zusman, 1996; Thiery and Kaimer, 2020), and *Herpetosiphon* sp. (Nett et al., 2006) are examples of predatory bacteria using this strategy.

The third approach is when cells enter the prey periplasm (Figure 1). The predator invades and develops in the periplasm of gram-negative bacteria (Hobot et al., 1984; Ferguson, 1990; Ferguson et al., 1992). Predator produces hydrolytic enzymes that promote penetration and damage the prey cell wall (Lerner et al., 2012). In parallel, the infected prey is destroyed once respiration comes to a halt and the outer membrane is damaged (Thomashow and Rittenberg, 1978). The invading predator attaches to the prey’s cytoplasmic membrane and initiates growth using the cytoplasm of the prey as a nutrient supply. The predator grows like a polynucleotide filament, the length of which is dependent on the prey size (Kessel and Shilo, 1976). In the end, filaments septate into individual attack-phase cells that grow a flagellum, induce the formation of pores in the cell wall and burst into the external medium to engage in another cycle (Fenton et al., 2010). Therefore, the predator kills the prey by ingesting its cytoplasm. *Bdellovibrionales* (Jurkevitch and Davidov, 2006), *Bdellovibrio bacteriovorus*, *Bacteriovorax marinus*, *Bacteriolyticum stolpii*, and *Peredibacter starrii*, use periplasmic predation (Sockett and Lambert, 2004; Pasternak et al., 2014). However, the mechanism used by predators to release their intracellular contents to achieve their original cell cycle is poorly described (Laloux, 2020).

Moreover, some predatory bacteria can utilize more than one hunting strategy, like *Bdellovibrio*, which employs the periplasmic and epibiotic strategy; also, *Myxobacteria* can use both epibiotic and wolfpack strategies.

The last category of predation includes all predators that penetrate the host cytoplasm (Figure 1). This approach is also known as the invasion of cytoplasm or diacytotic strategy (Moulder, 1985). After the penetration, the predator grows and divides inside the cytoplasm. *Daptobacter* is the only bacteria that employ this strategy (Guerrero et al., 1986), but no other study has been reported about this group. Among predatory *Actinomycetota*, a few investigations have highlighted strategies used in predation because they have not received the level of attention of competitors (Ibrahimi, 2020). Pérez et al. (2016) suggest that bacteria secreting secondary metabolites, including *Actinomycetota*, can attack their prey in groups, which is supported by the fact that they are social prokaryotes that form and develop in multicellular structures. Correspondingly, Kumbhar and Watve (2013) reported that antibiotic producers such as *Actinomycetota* are unable to use an endobiotic strategy and direct contact with the prey is not required. Kumbhar et al. (2014) showed that *Actinomycetota* are non-obligate epibiotic predators of diverse prey such as *S. aureus*, *E. coli*, *Bacillus* spp., *Pseudomonas aeruginosa*, and *Klebsiella* spp. Recently, a study by Zeng et al. (2021) demonstrated that *Streptomyces globisporus*, a predatory *Actinomycetota*, preyed on *Microcystis* through an epibiotic mode of predation. Overall, among the phylum *Actinomycetota*, only two genera, *Agromyces* and *Streptomyces*, are known to have an epibiotic predatory behavior against other bacterial species (Casida, 1983; Arcamone et al., 2000; Ibrahimi et al., 2020). All this information allows us to believe that the predatory *Actinomycetota* can hunt their prey through wolfpack and epibiotic strategies. Therefore, our understanding of *Actinomycetota* predation is still very fragmentary, including gaps in their mechanisms of predation. Understanding these mechanisms by *Actinomycetota* species is required for better knowledge and understanding of their effect on prey structure to develop a new strategy to control plant disease and multidrug-resistant pathogens.

## 3. Identity and diversity of small molecules produced from bacteria and *Actinomycetota* during predation lifestyles

Predatory bacteria represent a diversified collection of prokaryotic organisms that have the ability to consume other bacteria (Jurkevitch, 2007). While some of these bacteria act as solitary hunters, others are known to hunt in groups in a wide mixture before they attack their prey (Martin, 2002). This predatory strategy generally implicates the production of lytic enzymes and small bioactive compounds as predatory weapons (Rosenberg and Varon, 1984; Reichenbach and Höfle, 1993; Berleman and Kirby, 2009), while genome sequencing programs of these microorganisms have revealed the presence of very broad and varied secondary metabolites (Kiss et al., 2011). Therefore, extraction and purification of antimicrobial molecules from predatory bacteria have yielded the discovery of numerous novel molecules, as illustrated by jahnellamides (Plaza et al., 2013), salimyxins (Felder et al., 2013), cystomanamides (Etzbach et al., 2014), and precorallopyronin (Schäberle et al., 2015). It is confirmed by Xiao et al. (2011) that this variety of secondary metabolites is supposed to be involved in the death of the prey. It has been found that a defect in the production of these substances significantly affects predatory activity. In this section, all research relative to the results of chemical studies of compounds produced by predatory bacteria will be reviewed and critically analyzed in Table 2.

**TABLE 2:** Secondary metabolites from different predatory bacteria and their biological activity.

Predatory Bacteria	Product	Chemical Formula	Biological activity	References
*Aristabacter necator*	*Banegasine*	C_11_H_12_N_2_O_2_	Potentiate the antimicrobial activity of pyrrolnitrin	Cain et al., 2003
	Pyrrolnitrin	C_10_H_6_Cl_2_N_2_O_2_	Anti-fungal, anti-bacterial	
	*Maculosin*	C_14_H_16_N_2_O_3_	Potentiate the antimicrobial activity of pyrrolnitrin	
*Herpetosiphon sp.*	(+)-*O*-methylkolavelool	C_20_H_34_O	Anti-bacterial	Nakano et al., 2015
	Auriculamide	C_17_H_24_ClNO_4_	Antibiotic properties are still open	Schieferdecker et al., 2015
	Siphonazole	C_25_H_25_N_3_O_6_	Not reported	Nett et al., 2006
*M. xanthus DK1622*	Myxalamid B	C_25_H_40_NO_3_	Yeasts and Gram-positive bacteria	Gerth et al., 1983
	Myxochelin A	C_20_H_25_N_2_O_7_	Anti-bacterial and antitumoral activity	Kunze et al., 1989; Miyanaga et al., 2006; Krug et al., 2008
	Myxovirescin A1	C_34_H_60_NO_8_	Bactericidal	Gerth et al., 1982; Xiao et al., 2012
	Myxochromide A3	C_45_H_64_N_7_O_9_	Not reported	Korp et al., 2016
	DKxanthene-534	C_29_H_34_N_4_O_6_	Antioxidative activity	Meiser et al., 2006; Wenzel and Müller, 2009
	Myxoprincomide	C_45_H_76_N_10_O_16_	Not reported	Cortina et al., 2012
*Myxococcus fulvus Mxf50*	Myxopyronins A	C_23_H_31_NO_6_	Anti-bacterial activity	Kohl et al., 1983
	Myxopyronins B	C_24_H_33_NO_6_		
*Streptomyces althioticus Myxococcus* *Virescens* *M. xanthus* *Cystobacter fuscus*	Althiomycin	C_16_H_17_N_5_O_6_S_2_	Anti-bacterial activity	Fujimoto et al., 1970; Kunze et al., 1982
Myxobacterium	Gulmirecin B	C_22_H_34_O_9_	Anti-bacterial activity	Schieferdecker et al., 2014
Myxobacterium Pyxidicoccus fallax HKI 727	Gulmirecin A	C_27_H_42_O_1_	Anti-bacterial activity	Schieferdecker et al., 2014
*Cystobacter sp.*	Cystobactamids 919-2	C_25_H_29_N_3_O_7_	Anti-bacterial activity	Baumann et al., 2014
	Cystobactamids 919-1	C_46_H_45_N_7_O_14_	Anti-bacterial activity	Baumann et al., 2014
*Corallococcus coralloides*	Precorallopyronin A	C_29_H_39_NO_7_	Not reported	Korp et al., 2016
	Corallopyronins A	C_30_H_41_NO_7_	Block specifically eubacterial RNA polymerase	Irschik et al., 1985
	Corallopyronins C	C_30_H_41_NO_7_		
	Corallopyronins B	C_31_H_43_NO_7_		
*Lysobacter spp.*	Lysobactin	C_58_H_97_N_15_O_17_	Anti-bacterial activity	O’Sullivan et al., 1988
*Myxobacterium* *Enhygromyxa salina*	Salimyxin A	C_21_H_30_O	Anti-bacterial activity	Felder et al., 2013
	Salimyxin B	C_21_H_32_O		

Traditionally, *Actinomycetota* species are renowned for their excellent potential to produce secondary metabolites and antibiotics compounds (Hoshino et al., 2015; Ait Barka et al., 2016; Ouchari et al., 2019; Ibrahimi et al., 2020; Korichi et al., 2021). These molecules are produced to antagonize the growth of surrounding microbes (Ortiz-Ortiz et al., 2013). To date, up to 33 new secondary metabolites have been successfully isolated from 12 *Actinomycetota* through the co-culture (Hoshino et al., 2019). With their novel roles as predatory microbes, the diversity and identity of molecules produced during predation strategies should be considered an untapped source of biomolecules. A further co-culturing of predator and prey monitored for 15 days induced an increase in the total amount of methylated fatty acids biomarker of the predatory *Actinomycetota* responsible for the predation process (Ibrahimi et al., 2019). Therefore, secondary metabolites engaged in the facultative predation and antagonistic mechanism are likely to differ, from the *Actinomycetota* strain, the origin of habitat, or the ecology of isolated strains (McBride and Zusman, 1996; Jurkevitch, 2007; Octaviana, 2021).

Predatory *Actinomycetota* are mostly isolated from marine and soil environments (Kumbhar et al., 2014; Ibrahimi et al., 2020; Baig et al., 2021). They exhibit a wide range of predatory activities against diverse bacteria (Kumbhar et al., 2014; Ibrahimi et al., 2020; Baig et al., 2021). This is explained by the bioactive compound that they produce, which possess a range of antimicrobial activities. These molecules are used as a weapon by predatory *Actinomycetota* to kill their prey (Kumbhar and Watve, 2013). Recently, Baig et al. (2021) demonstrated a strong correlation between predation and enzyme inhibition, particularly trypsin and chymotrypsin inhibition, in which predatory *Actinomycetota* were found to release more enzymes in the presence of prey. On this basis, it has become apparent that *Actinomycetota* can exhibit the great potential to produce antibiotics and enzymes during predation. Also, it should be noted that, to date, no compounds produced by predatory *Actinomycetota* during predation behavior have been elucidated. Therefore, it will be interesting to conduct further studies to isolate new predatory *Actinomycetota* as well as the extraction and identification of their molecules involved in predation lifestyle.

## 4. Predatory *Actinomycetota* as an eco-friendly and promising tool in agricultural and environmental sustainability

### 4.1. Plant pathogens

Recent years have been marked by an expanding array of virulent infectious diseases caused by pests which are increasingly recognized as presenting a worldwide threat to food security (Olsen et al., 2011; Bosso et al., 2015; Hartmann, 2022). In addition, the extensive use of agrochemicals molecules has led to the development of bacterial resistance, causing significant risks to the environment and human health (Ebele Mbachu et al., 2022). Consequently, providing food for the world’s population without disrupting the environmental balance is becoming eminent (Pandit et al., 2022). It is highly recommended to provide sustainable solutions for agriculture (Jamiołkowska, 2020). Microbes are an alternative to agrochemical molecules like synthetic pesticides for controlling plant pathogens (Elnahal et al., 2022). Different microorganisms are used as biocontrol agents, such as bacteria, fungi, and *Actinomycetota* (Table 3). Direct antagonism and predation are the possible modes of action of biocontrol agents to eliminate plant parasites (Ebele Mbachu et al., 2022).

**TABLE 3:** Predatory bacteria application.

Predatory bacteria	Pathogen	Application	References
*Bacillus*	Bacteria, fungi and oomycetes	Biocontrol against phytopathogens, including bacteria, fungi and oomycetes	Ongena and Jacques, 2008
	*Fusarium fungi*	Biocontrol agent of *Fusarium* head blight of wheat	Schisler et al., 2002
	Phytophthora species such as *P. capsica*	Biocontrol agent for *P. capsici* pathogenic fungi	Douillet, 2003
*Bacteriovorax* spp.	*Vibrio vulnificus* and *Vibrio parahaemolyticus*	Reduced *Vibrio* sp. populations	Richards et al., 2012, 2013
*Bdellovibrio bacteriovorus*	*Pseudomonas aeruginosa* and *Staphylococcus aureus*	Reduced *S. aureus* (periplasmic on Gram-negative and epibiotic on Gram-positive).	Iebba et al., 2014
	*Staphylococcus aureus*	Reduced biofilm formation	Monnappa et al., 2014
	Multidrug resistant Gram-negative bacteria	Reduced biofilm formation	Kadouri et al., 2013
	Gram-negative foodborne pathogens and spoilage bacteria	Lysed foodborne and spoilage bacteria	Fratamico and Whiting, 1995
	*Salmonella enterica Klebsiella pneumoniae Escherichia coli Enterobacter*	Biocontrol agent to treat urban wastewater treatment	Jafarian et al., 2020
	Microbial biomass in sludge	Alteration of the microbial community composition of activated sludge flocs and granules	Feng et al., 2017
*Bdellovibrio bacteriovorus* *Lysobacter enzymogenes*	*Pseudomonas tolaasii*	Reduced bacterial cells *in vitro* and blotch severity on pilei of mushroom at post-harvest	Saxon et al., 2014
	Shrimp pathogens *V. cholerae*	Biocontrol agent in freshwater farming industry as a biological control	Cao et al., 2015, 2019
	*Alcaligenes, campylobacter, Erwinia, Escherichia, Helicobacter, Pseudomonas, Legionella, and Shigella*	Biocontrol agent	Markelova, 2010
	*Escherichia coli*	Lyse gram-negative foodborne pathogenic and spoilage bacteria Novel antibody-modulating tools	Fratamico and Whiting, 1995; Bratanis et al., 2017; Bratanis and Lood, 2019
	*Bipolaris sorokiniana*	Role in fungal plant disease control	Li et al., 2008
*Myxobacteria*	*Cylindrocarpon* spp., *Fusarium oxysporum*. sp. *apii, Phytophthora capsici*, *Pythium ultimum*, *Rhizoctonia* spp., *Sclerotinia minor*…	Plant pathogenic fungi	Bull et al., 2002
*Pseudomonas fluorescens*	*Tobacco necrosis* virus	Biocontrol agent against phytopathogens	Maurhofer et al., 1998
*Micavibrio aeruginosavorus and B. bacteriovorus*	Gram-negative *bacteria*: *Pseudomonas aeruginosa* and *Escherichia coli*	Biocontrol agent against phytogenic bacteria	Dashiff et al., 2011
*Streptomyces griseoflavus EMM111 Streptomyces coelicoflavus EMM112 Streptomyces mutabilis EMM183 Streptomyces champavatii EMM184*	*Micrococcus luteus*, *Staphylococcus aureus* (native and methicillin-resistant) *Escherichia coli* (native and ampicillin-resistant)	Biocontrol agent against multidrug-resistant bacteria	Ibrahimi et al., 2019, 2020
*Brevibacterium, Glutamicibacter, Micromonospora, Nocardiopsis, Rhodococcus and Rothia*	*Acetobacter pasteurianus Alcaligenes fecalis Bacillus subtilis Enterobacter fecalis, Escherichia coli Klebsiella pneumoniae Micrococcus luteus Mycobacterium smegmatis Proteus vulgaris Pseudomonas aeruginosa Salinicoccus roseus Salmonella enterica Serratia marcescens Staphylococcus aureus*	Biocontrol agent against pathogenic bacteria	Baig et al., 2021

Predatory bacteria can be used as alternative applications in biological control (Olanya and Lakshman, 2015; Swain et al., 2017). Most of the Bacillus strains can control the plant pathogen as *Fusarium* fungi (Ongena and Jacques, 2008). *Bdellovibrio* also attacks a large variety of different plant pathogens (Baer et al., 2000; Jurkevitch et al., 2000; Dwidar et al., 2012). In addition, a very promising result in the control by *Bdellovibrio* of *Pseudomonas glycinea* blight of soybean was demonstrated by Scherff (1973). Recently, Ye et al. (2020) demonstrated the use of the Myxobacterium *Corallococcus coralloides* to control cucumber *Fusarium* wilt by migrating to the plant root and regulating the soil microbial community.

In the case of *Actinomycetota*, several investigations showed their successful ability to function as biocontrol agents against plant pathogens (Goudjal et al., 2014; Braga et al., 2016; Ebrahimi-Zarandi et al., 2022). The co-cultivation of *Actinomycetota* with other microorganisms generates several new secondary metabolites, which are not present during pure culture conditions (Scherlach and Hertweck, 2009; Sung et al., 2017; Wakefield et al., 2017; Shin et al., 2018; Vikeli et al., 2019; Yu M. et al., 2019). Indeed, the production of secondary metabolites in co-culture is enhanced by competitive or antagonistic interactions (Ibrahimi et al., 2020). For example, the co-cultivation of the *Streptomyces coelicolor* with the agricultural pathogen *Aspergillus niger* has activated the actinorhodin silent pathway in *Actinomycetota* (Wu et al., 2015).

Prior research suggests that *Actinomycetota* in co-culture can inhibit pathogens’ growth, decrease, and degrade toxins. For example, a recent investigation demonstrated that coculturing *Streptomyces roseolus* with the phytopathogen *Aspergillus flavus* could reduce the contamination generated by the mycotoxin aflatoxin B1, which is produced by *A. flavus* (Caceres et al., 2018). In addition, several *Streptomyces* strains showed the ability to inhibit *Aspergillus flavus* growth and decrease and degrade mycotoxin (Verheecke et al., 2015; Campos-Avelar et al., 2021). Another field that seems promising for the use of *Actinomycetota* in agriculture is their function as eco-friendly biofertilizers since they are involved in nutrient management, soil quality, decomposing of organic matter, enhancing plant growth promoting, recycling organic residues, and activating plant immune responses (Shivlata and Satyanarayana, 2017; Boubekri et al., 2022; Ebrahimi-Zarandi et al., 2022).

Using predatory *Actinomycetota* in co-culture may be a potential biocontrol agent and biofertilizer used in agriculture. Indeed, the plant can benefit on several different levels. It was reported in the literature that the co-cultivation of *Streptomyces* with the phytopathogenic fungus *Sclerotinia sclerotiorum* induces the deformation and fragmentation of the fungal mycelium through the production of hydrolytic enzymes and secondary metabolites (Liu et al., 2019). In parallel, the same study linked the promotion of plant growth through the solubilization of inorganic phosphate and the production of 1-aminocyclopropane-1-carboxylate deaminase and indole acetic acid by the *Actinomycetota* (Liu et al., 2019).

The predation of gram-negative and gram-positive bacteria by *Actinomycetota* may enhance their potential application for the biocontrol of foodborne and plant pathogens (Ibrahimi et al., 2019, 2020). Thus, predatory *Actinomycetota* can provide an ecologically sustainable solution for agricultural farming. This is explained by the fact that they will not increase the accumulation of antibiotics in the environment, which may generate antibiotic resistance (Pérez et al., 2020).

### 4.2. Cyanobacterial bloom

Cyanobacterial bloom can produce toxic molecules called cyanotoxins in freshwater (Saraf et al., 2018). The presence of such molecules can affect the functionality of ecosystems and water quality for recreation, drinking water, fisheries, and agriculture (O’Neil et al., 2012; Corbel et al., 2014). In the agriculture field, several publications have appeared in recent years documenting the bioaccumulation of cyanotoxins in plants used for human and animal food (Corbel et al., 2014; Machado et al., 2017; Ai et al., 2020; Melaram et al., 2022). Consequently, several physical, chemical, and biological strategies were deployed to control cyanobacterial bloom (Jia et al., 2018; Sun et al., 2018; Yu Y. et al., 2019; El Amrani Zerrifi et al., 2020). Although the physic-chemical techniques represent a high cost, the risk of contamination and toxicity to humans has limited their general use (Moreira et al., 2014). Whereas biological techniques involving microorganisms have attracted researchers for their promising eco-friendly tools and high potential (Yu Y. et al., 2019).

One of the most promising aspects is the use of microbes of predatory bacteria as a control agent of cyanobacterial blooms. However, despite some initial promising discoveries, this field has been almost completely ignored. The first predatory bacteria able to lyse various species of cyanobacteria was first reported in 1967 (Shilo, 1967). Since then, numerous strains of lytic gliding bacteria, mainly members of the *Myxobacteria* and *Cytophaga* groups, have been isolated (Daft and Stewart, 1971; Granhall and Berg, 1972; Gumbo et al., 2008) and lysed cyanobacteria cells by attachment and secretion of diffusible lytic substances. These bacteria produce a variety of different exoenzymes capable of hydrolyzing the cyanobacterial cell wall (Sudo and Dworkin, 1972; Gnosspelius, 1978). Subsequently, Rashidan and Bird (2001) isolated two strains of *Cytophaga* sp., with lytic activity on different cyanobacteria with a restricted host range. The same study showed that the lysis of cyanobacteria by predatory bacteria may be an important factor in their population dynamics in lakes and may contribute to the prevention or the sudden disappearance of cyanobacteria blooms and draw attention to the possibilities of using host-specific lytic bacteria in biological control of harmful cyanobacterial blooms (Rashidan and Bird, 2001).

Furthermore, *Bdellovibrio* and *Myxococcus* have received a lot of investigation as predators of cyanobacterial bloom (Bauer and Forchhammer, 2021). In contrast, the use of *Actinomycetota* as a predator is still broadly uncharacterized. However, recently Zeng et al. (2021) demonstrated the use of a predatory *Actinomycetota* to face harmful cyanobacterial algal blooms. The research demonstrated that *Streptomyces globisporus* could predate *Microcystis aeruginosa via* cell-to-cell contact with high algicidal activity (Zeng et al., 2021). The present findings confirm the use of predatory *Actinomycetota* as promising eco-friendly tools to combat harmful cyanobacteria blooms. Also, further investigations are highly recommended to assess the predation toward cyanobacteria. In addition, it is necessary to recognize the predatory *Actinomycetota*-cyanobacteria ratio due to their important role as a key to achieving effective lysis of cyanobacteria.

## 5. Future perspectives and conclusion

Nowadays, one of the most alarming world wild problems is the increase of plant diseases caused by pathogenic bacteria, which causes great economic, environmental, and human health damage. Therefore, finding alternative and sustainable solutions to confront these pathogens represent one of the biggest challenges. In this review, we suggest the use of predatory *Actinomycetota* as an effective biocontrol agent. Predation is an important cause of mortality and determines the structure and activity of microbial communities in both terrestrial and aquatic ecosystems, with a complex process involving several components such as prey finding, recognition, consumption, and digestion. The literature has concentrated largely on the presence of predatory *Actinomycetota* and their effective activity against a panel of pathogenic microorganisms but has not examined the mechanisms of the prey lysis. Indeed, a good knowledge of the predation mechanism used by predatory *Actinomycetota* is essential to improve the efficiency of the lysis of the prey cells and predation performance. The application of predatory *Actinomycetota* in large-scale systems and field experiments must be examined to determine if there will be ecological consequences. Furthermore, it is suggested that research evaluating predators’ efficiency should include molecular viability assays, such as EMAqPCR and EMA-Illumina, to determine the efficacy of treatment. Finally, during this review, we noticed that antibiotics produced during the process of predation by predatory *Actinomycetota* are demonstrated but have not yet been identified. Consequently, it seems advisable to discover such compounds. In the future, actinobacterial predation could be a new approach to control plant pathogen, cyanobacterial blooms; however, intensive research efforts are required to pursue this aim.

## Author contributions

MI wrote the manuscript. YO and MJ refining and critical reading of the manuscript. LL, SL, and MH revised the manuscript. All authors contributed to the manuscript and approved the submitted version.

## References

[B1] AbdElgawad H. Abuelsoud W. Madany M. M. Selim S. Zinta G. Mousa A. S. (2020). Actinomycetes enrich soil rhizosphere and improve seed quality as well as productivity of legumes by boosting nitrogen availability and metabolism. *Biomolecules* 10:1675. 10.3390/biom10121675 33333896 PMC7765327

[B2] Ai Y. Lee S. Lee J. (2020). Drinking water treatment residuals from cyanobacteria bloom-affected areas: Investigation of potential impact on agricultural land application. *Sci. Total Environ.* 706:135756. 10.1016/j.scitotenv.2019.135756 31940734

[B3] Ait Barka E. A. Vatsa P. Sanchez L. Gaveau-Vaillant N. Jacquard C. Klenk H.-P. (2016). Taxonomy, physiology, and natural products of actinobacteria. *Microbiol. Mol. Biol. Rev.* 80 1–43. 10.1128/MMBR.00019-15 26609051 PMC4711186

[B4] Alvarez A. Saez J. M. Costa J. S. D. Colin V. L. Fuentes M. S. Cuozzo S. A. (2017). Actinobacteria: Current research and perspectives for bioremediation of pesticides and heavy metals. *Chemosphere* 166 41–62. 10.1016/j.chemosphere.2016.09.070 27684437

[B5] Arcamone F. Cassinelli G. Fantini G. Grein A. Orezzi P. Pol C. (2000). Adriamycin, 14-Hydroxydaunomycin, a new antitumor antibiotic from *S. peucetius* var. *caesius*. *Biotechnol. Bioeng.* 67 704–713. 10.1002/(SICI)1097-0290(20000320)67:6<704::AID-BIT8>3.0.CO;2-L 10699851

[B6] Baer M. L. Ravel J. Chun J. Hill R. T. Williams H. N. (2000). A proposal for the reclassification of *Bdellovibrio stolpii* and *Bdellovibrio starrii* into a new genus, *Bacteriovorax* gen. nov. as *Bacteriovorax stolpii* comb. nov. and *Bacteriovorax starrii* comb. nov., respectively. *Int. J. Syst. Evol. Microbiol.* 50 219–224. 10.1099/00207713-50-1-219 10826807

[B7] Baig U. Dahanukar N. Shintre N. Holkar K. Pund A. Lele U. (2021). Phylogenetic diversity and activity screening of cultivable actinobacteria isolated from marine sponges and associated environments from the western coast of India. *Access Microbiol.* 3:000242. 10.1099/acmi.0.000242 34712902 PMC8549387

[B8] Baltz R. H. (2019). Natural product drug discovery in the genomic era: Realities, conjectures, misconceptions, and opportunities. *J. Ind. Microbiol. Biotechnol.* 46 281–299. 10.1007/s10295-018-2115-4 30484124

[B9] Banning E. C. (2010). *Biology and potential biogeochemical impacts of novel predatory flavobacteria*. DTIC Document, Massachusetts Institute of Technology: Cambridge, MA.

[B10] Batinovic S. Rose J. J. Ratcliffe J. Seviour R. J. Petrovski S. (2021). Cocultivation of an ultrasmall environmental parasitic bacterium with lytic ability against bacteria associated with wastewater foams. *Nat. Microbiol.* 6 703–711. 10.1038/s41564-021-00892-1 33927381

[B11] Bauer A. Forchhammer K. (2021). Bacterial predation on cyanobacteria. *Microb. Physiol.* 31 99–108. 10.1159/000516427 34010833

[B12] Baumann S. Herrmann J. Raju R. Steinmetz H. Mohr K. I. Hüttel S. (2014). Cystobactamids: Myxobacterial topoisomerase inhibitors exhibiting potent antibacterial activity. *Angew. Chem. Int. Ed.* 53 14605–14609. 10.1002/anie.201409964 25510965

[B13] Be’Er A. Zhang H. P. Florin E.-L. Payne S. M. Ben-Jacob E. Swinney H. L. (2009). Deadly competition between sibling bacterial colonies. *Proc. Natl. Acad. Sci. U.S.A.* 106 428–433. 10.1073/pnas.0811816106 19129489 PMC2626719

[B14] Bentley S. D. Chater K. F. Cerdeño-Tárraga A.-M. Challis G. L. Thomson N. R. James K. D. (2002). Complete genome sequence of the model actinomycete *Streptomyces coelicolor* A3 (2). *Nature* 417:141. 12000953 10.1038/417141a

[B15] Berleman J. E. Kirby J. R. (2009). Deciphering the hunting strategy of a bacterial wolfpack. *FEMS Microbiol. Rev.* 33 942–957. 10.1111/j.1574-6976.2009.00185.x 19519767 PMC2774760

[B16] Berleman J. E. Chumley T. Cheung P. Kirby J. R. (2006). Rippling is a predatory behavior in *Myxococcus xanthus*. *J. Bacteriol.* 188 5888–5895. 10.1128/JB.00559-06 16885457 PMC1540073

[B17] Bor B. Collins A. J. Murugkar P. P. Balasubramanian S. To T. T. Hendrickson E. L. (2020). Insights obtained by culturing Saccharibacteria with their bacterial hosts. *J. Dent. Res.* 99 685–694. 10.1177/0022034520905792 32075512 PMC7243422

[B18] Bosso L. Scelza R. Testa A. Cristinzio G. Rao M. A. (2015). Depletion of pentachlorophenol contamination in an agricultural soil treated with *Byssochlamys nivea*, *Scopulariopsis brumptii* and urban waste compost: A laboratory microcosm study. *Water Air Soil Pollut.* 226:183. 10.1007/s11270-015-2436-0

[B19] Boubekri K. Soumare A. Mardad I. Lyamlouli K. Ouhdouch Y. Hafidi M. (2022). Multifunctional role of actinobacteria in agricultural production sustainability: A review. *Microbiol. Res.* 261:127059. 10.1016/j.micres.2022.127059 35584559

[B20] Braga R. M. Dourado M. N. Araújo W. L. (2016). Microbial interactions: Ecology in a molecular perspective. *Braz. J. Microbiol.* 47 86–98. 10.1016/j.bjm.2016.10.005 27825606 PMC5156507

[B21] Bratanis E. Lood R. (2019). A novel broad-spectrum elastase-like serine protease from the predatory bacterium *Bdellovibrio bacteriovorus* facilitates elucidation of site-specific IgA glycosylation pattern. *Front. Microbiol.* 10 971. 10.3389/fmicb.2019.00971 31130941 PMC6510308

[B22] Bratanis E. Andersson T. Lood R. Bukowska-Faniband E. (2020). Biotechnological potential of *Bdellovibrio* and like organisms and their secreted enzymes. *Front. Microbiol.* 11:662. 10.3389/fmicb.2020.00662 32351487 PMC7174725

[B23] Bratanis E. Molina H. Naegeli A. Collin M. Lood R. (2017). BspK, a serine protease from the predatory bacterium *Bdellovibrio bacteriovorus* with utility for analysis of therapeutic antibodies. *Appl. Environ. Microbiol.* 83 e03037-16. 10.1128/AEM.03037-16 27940543 PMC5288813

[B24] Bull C. T. Shetty K. G. Subbarao K. V. (2002). Interactions between myxobacteria, plant pathogenic fungi, and biocontrol agents. *Plant Dis.* 86 889–896. 10.1094/PDIS.2002.86.8.889 30818644

[B25] Caceres I. Snini S. P. Puel O. Mathieu F. (2018). Streptomyces roseolus, a promising biocontrol agent against *Aspergillus flavus*, the main aflatoxin B1 producer. *Toxins* 10:442. 10.3390/toxins10110442 30380704 PMC6267218

[B26] Cain C. C. Lee D. Waldo R. H. Henry A. T. Casida E. J. Wani M. C. (2003). Synergistic antimicrobial activity of metabolites produced by a nonobligate bacterial predator. *Antimicrob. Agents Chemother.* 47 2113–2117. 10.1128/AAC.47.7.2113-2117.2003 12821455 PMC161883

[B27] Campos-Avelar I. Colas De La Noue A. Durand N. Cazals G. Martinez V. Strub C. (2021). *Aspergillus flavus* growth inhibition and aflatoxin B1 decontamination by *Streptomyces* isolates and their metabolites. *Toxins* 13:340. 10.3390/toxins13050340 34066812 PMC8151643

[B28] Cao H. An J. Zheng W. He S. (2015). *Vibrio cholerae* pathogen from the freshwater-cultured whiteleg shrimp *Penaeus vannamei* and control with *Bdellovibrio bacteriovorus*. *J. Invertebr. Pathol.* 130 13–20. 10.1016/j.jip.2015.06.002 26146226

[B29] Cao H. Wang H. Yu J. An J. Chen J. (2019). Encapsulated *Bdellovibrio* powder as a potential bio-disinfectant against whiteleg shrimp-pathogenic vibrios. *Microorganisms* 7:244. 10.3390/microorganisms7080244 31394792 PMC6722716

[B30] Casida L. E. (1980). Bacterial predators of *Micrococcus luteus* in soil. *Appl. Environ. Microbiol.* 39 1035–1041. 10.1128/aem.39.5.1035-1041.1980 16345566 PMC291471

[B31] Casida L. E. (1983). Interaction of *Agromyces ramosus* with other bacteria in soil. *Appl. Environ. Microbiol.* 46 881–888. 10.1128/aem.46.4.881-888.1983 16346402 PMC239483

[B32] Casida L. E. (1988). Minireview: Nonobligate bacterial predation of bacteria in soil. *Microb. Ecol.* 15 1–8. 10.1007/BF02012948 24202859

[B33] Casida L. E. (1992). Competitive ability and survival in soil of *Pseudomonas* strain 679-2, a dominant, nonobligate bacterial predator of bacteria. *Appl. Environ. Microbiol.* 58 32–37. 10.1128/aem.58.1.32-37.1992 16348631 PMC195168

[B34] Casida L. E. Jr. (1982). *Ensifer adhaerens* gen. nov., sp. nov.: A bacterial predator of bacteria in soil. *Int. J. Syst. Evol. Microbiol.* 32 339–345. 10.1099/00207713-32-3-339

[B35] Castelle C. J. Banfield J. F. (2018). Major new microbial groups expand diversity and alter our understanding of the tree of life. *Cell* 172 1181–1197. 10.1016/j.cell.2018.02.016 29522741

[B36] Cavallo F. M. Jordana L. Friedrich A. W. Glasner C. van Dijl J. M. (2021). *Bdellovibrio bacteriovorus*: A potential ‘living antibiotic’ to control bacterial pathogens. *Crit. Rev. Microbiol.* 47 630–646. 10.1080/1040841X.2021.1908956 33934682

[B37] Chernyakovskaya T. F. Dobrovol’skaya T. G. Bab’eva I. P. (2004). The ability of saprotrophic bacteria isolated from natural habitats to lyse yeasts. *Microbiology* 73 482–484. 10.1023/B:MICI.0000036995.67735.df 15521184

[B38] Collinge D. B. Sarrocco S. (2022). Transgenic approaches for plant disease control: Status and prospects 2021. *Plant Pathol.* 71 207–225. 10.1111/ppa.13443

[B39] Corbel S. Mougin C. Bouaïcha N. (2014). Cyanobacterial toxins: Modes of actions, fate in aquatic and soil ecosystems, phytotoxicity and bioaccumulation in agricultural crops. *Chemosphere* 96 1–15. 10.1016/j.chemosphere.2013.07.056 24012139

[B40] Cortina N. S. Krug D. Plaza A. Revermann O. Müller R. (2012). Myxoprincomide: A natural product from *Myxococcus xanthus* discovered by comprehensive analysis of the secondary metabolome. *Angew. Chem. Int. Ed.* 51 811–816. 10.1002/anie.201106305 22162209

[B41] Cotter T. W. Thomashow M. F. (1992). A conjugation procedure for *Bdellovibrio bacteriovorus* and its use to identify DNA sequences that enhance the plaque-forming ability of a spontaneous host-independent mutant. *J. Bacteriol.* 174 6011–6017. 10.1128/jb.174.19.6011-6017.1992 1400153 PMC207665

[B42] Cross K. L. Campbell J. H. Balachandran M. Campbell A. G. Cooper C. J. Griffen A. (2019). Targeted isolation and cultivation of uncultivated bacteria by reverse genomics. *Nat. Biotechnol.* 37 1314–1321. 10.1038/s41587-019-0260-6 31570900 PMC6858544

[B43] Curds C. R. (1982). The ecology and role of protozoa in aerobic sewage treatment processes. *Annu. Rev. Microbiol.* 36 27–28. 10.1146/annurev.mi.36.100182.000331 6816137

[B44] Daft M. J. Stewart W. D. P. (1971). Bacterial pathogens of freshwater blue-green algae. *New Phytol.* 70 819–829. 10.1111/j.1469-8137.1971.tb02582.x

[B45] Dashiff A. Junka R. A. Libera M. Kadouri D. E. (2011). Predation of human pathogens by the predatory bacteria *Micavibrio aeruginosavorus* and *Bdellovibrio bacteriovorus*. *J. Appl. Microbiol.* 110 431–444. 10.1111/j.1365-2672.2010.04900.x 21114596

[B46] Davidov Y. Huchon D. Koval S. F. Jurkevitch E. (2006). A new α-proteobacterial clade of *Bdellovibrio*-like predators: Implications for the mitochondrial endosymbiotic theory. *Environ. Microbiol.* 8 2179–2188. 10.1111/j.1462-2920.2006.01101.x 17107559

[B47] Douillet P. (2003). *Strains of Bacillus for biological control of pathogenic fungi*. Google Patents. Available online at: https://www.google.com/patents/US6589524 (accessed June 16, 2017).

[B48] Druga B. Suteu D. Rosca-Casian O. Parvu M. Dragos N. (2011). Two novel *Alliin lyase* (alliinase) genes from twisted-leaf garlic (*Allium obliquum*) and mountain garlic (*Allium senescens* ssp. *montanum*). *Notulae Bot. Horti Agrobot. Cluj Napoca* 39:293. 10.15835/nbha3926355

[B49] Dwidar M. Monnappa A. K. Mitchell R. J. (2012). The dual probiotic and antibiotic nature of *Bdellovibrio bacteriovorus*. *BMB Rep.* 45 71–78. 10.5483/BMBRep.2012.45.2.71 22360883

[B50] Ebele Mbachu A. Obianom A. O. Ogbonna U. S. Mbachu N. A. Abumchukwu Okoli F. (2022). Mode of attack of microbiological control agents against plant pathogens for sustainable agriculture and food security. *Asian J. Agric. Hortic. Res*. 9, 1–16. 10.9734/ajahr/2022/v9i130132

[B51] Ebrahimi-Zarandi M. Saberi Riseh R. Tarkka M. T. (2022). Actinobacteria as effective biocontrol agents against plant pathogens, an overview on their role in eliciting plant defense. *Microorganisms* 10:1739. 10.3390/microorganisms10091739 36144341 PMC9500821

[B52] El Amrani Zerrifi S. El Khalloufi F. Mugani R. El Mahdi R. Kasrati A. Soulaimani B. (2020). Seaweed essential oils as a new source of bioactive compounds for cyanobacteria growth control: Innovative ecological biocontrol approach. *Toxins* 12:527. 10.3390/toxins12080527 32824610 PMC7472222

[B53] Elnahal A. S. El-Saadony M. T. Saad A. M. Desoky E. S. M. El-Tahan A. M. Rady M. M. (2022). The use of microbial inoculants for biological control, plant growth promotion, and sustainable agriculture: A review. *Eur. J. Plant Pathol.* 162 759–792. 10.1007/s10658-021-02393-7

[B54] Etzbach L. Plaza A. Garcia R. Baumann S. Müller R. (2014). Cystomanamides: Structure and biosynthetic pathway of a family of glycosylated lipopeptides from myxobacteria. *Org. Lett.* 16 2414–2417. 10.1021/ol500779s 24735013

[B55] Ezzedine J. A. Jacas L. Desdevises Y. Jacquet S. (2020). *Bdellovibrio* and like organisms in Lake Geneva: An unseen elephant in the room? *Front. Microbiol.* 11:98. 10.3389/fmicb.2020.00098 32117128 PMC7034301

[B56] Felder S. Kehraus S. Neu E. Bierbaum G. Schäberle T. F. König G. M. (2013). Salimyxins and enhygrolides: Antibiotic, sponge-related metabolites from the obligate marine myxobacterium *Enhygromyxa salina*. *ChemBioChem* 14 1363–1371. 10.1002/cbic.201300268 23794290

[B57] Feng S. Tan C. H. Constancias F. Kohli G. S. Cohen Y. Rice S. A. (2017). Predation by *Bdellovibrio bacteriovorus* significantly reduces viability and alters the microbial community composition of activated sludge flocs and granules. *FEMS microbiol. Ecol.* 93:fix020. 10.1093/femsec/fix020 28334102

[B58] Fenton A. K. Lambert C. Wagstaff P. C. Sockett R. E. (2010). Manipulating each MreB of *Bdellovibrio bacteriovorus* gives diverse morphological and predatory phenotypes. *J. Bacteriol.* 192 1299–1311. 10.1128/JB.01157-09 20023029 PMC2820843

[B59] Ferguson S. J. (1990). Periplasm underestimated. *Trends Biochem. Sci.* 15:377. 10.1016/0968-0004(90)90234-3 2251728

[B60] Ferguson S. J. Mohan S. Dow C. Cole J. A. (1992). *The periplasm.* New York, NY: Cambridge University Press.

[B61] Fratamico P. M. Cooke P. H. (1996). Isolation of *Bdellovibrio*s that prey on *Escherichia coli* O157: H7 and *Salmonella* species and application for removal of prey from stainless steel surfaces1. *J. Food Safety* 16 161–173. 10.1111/j.1745-4565.1996.tb00157.x

[B62] Fratamico P. M. Whiting R. C. (1995). Ability of *Bdellovibrio bacteriovorus* 109J to lyse gram-negative food-borne pathogenic and spoilage bacteria. *J. Food Prot.* 58 160–164. 10.4315/0362-028X-58.2.160 31121669

[B63] Fujimoto H. Kinoshita T. Suzuki H. Umezawa H. (1970). Studies on the mode of action of althiomycin. *J. Antibiot.* 23 271–275. 10.7164/antibiotics.23.271 4917791

[B64] Furness E. Whitworth D. E. Zwarycz A. (2020). “Predatory interactions between myxobacteria and their prey,” in *The ecology of predation at the microscale*, eds Jurkevitch E. Mitchell R. (Cham: Springer), 1–36. 10.1007/978-3-030-45599-6_1

[B65] Furusawa G. Yoshikawa T. Yasuda A. Sakata T. (2003). Algicidal activity and gliding motility of *Saprospira* sp. SS98-5. *Can. J. Microbiol.* 49 92–100. 10.1139/w03-017 12718397

[B66] Germida J. J. Casida L. E. (1983). Ensifer adhaerens predatory activity against other bacteria in soil, as monitored by indirect phage analysis. *Appl. Environ. Microbiol.* 45 1380–1388. 10.1128/aem.45.4.1380-1388.1983 16346275 PMC242466

[B67] Gerth K. Irschik H. Reichenbach H. Trowitzsch W. (1982). The myxovirescins, a family of antibiotics from *Myxococcus virescens* (Myxobacterales). *J. Antibiot.* 35 1454–1459. 10.7164/antibiotics.35.1454 6819280

[B68] Gerth K. Jansen R. Reifenstahl G. Höfle G. Irschik H. Kunze B. (1983). The myxalamids, new antibiotics from *Myxococcus xanthus* (Myxobacterales). *J. Antibiot.* 36 1150–1156. 10.7164/antibiotics.36.1150 6415031

[B69] Gnosspelius G. (1978). Purification and properties of an extracellular protease from *Myxococcus virescens*. *J. Bacteriol.* 133 17–25. 10.1128/jb.133.1.17-25.1978 22536 PMC221971

[B70] Gonzalez J. M. Sherr E. B. Sherr B. F. (1990). Size-selective grazing on bacteria by natural assemblages of estuarine flagellates and ciliates. *Appl. Environ. Microbiol.* 56 583–589. 10.1128/aem.56.3.583-589.1990 2107794 PMC183390

[B71] Goudjal Y. Toumatia O. Yekkour A. Sabaou N. Mathieu F. Zitouni A. (2014). Biocontrol of *Rhizoctonia solani* damping-off and promotion of tomato plant growth by endophytic actinomycetes isolated from native plants of Algerian Sahara. *Microbiol. Res.* 169 59–65. 10.1016/j.micres.2013.06.014 23920229

[B72] Granhall U. Berg B. (1972). Antimicrobial effects of *Cellvibrio* on blue-green algae. *Arch. Microbiol.* 84 234–242. 10.1007/BF00425201 4626336

[B73] Guerrero R. Pedrós-Alió C. Esteve I. Mas J. Chase D. Margulis L. (1986). Predatory prokaryotes: Predation and primary consumption evolved in bacteria. *Proc. Natl. Acad. Sci. U.S.A.* 83 2138–2142. 10.1073/pnas.83.7.2138 11542073 PMC323246

[B74] Gumbo J. R. Ross G. Cloete T. E. (2010). The isolation and identification of predatory bacteria from a *Microcystis* algal bloom. *Afr. J. Biotechnol.* 9 663–671. 10.5897/AJB09.834

[B75] Gumbo R. J. Ross G. Cloete E. T. (2008). Biological control of *Microcystis* dominated harmful algal blooms. *Afr. J. Biotechnol.* 7 4765–4773.

[B76] Hartmann F. E. (2022). Using structural variants to understand the ecological and evolutionary dynamics of fungal plant pathogens. *New Phytol.* 234 43–49. 10.1111/nph.17907 34873717

[B77] Hashizume H. Hirosawa S. Sawa R. Muraoka Y. Ikeda D. Naganawa H. (2004). Tripropeptins, novel antimicrobial agents produced by *Lysobacter* sp. *J. Antibiot.* 57 52–58. 10.7164/antibiotics.57.52 15032486

[B78] Hobley L. Lerner T. R. Williams L. E. Lambert C. Till R. Milner D. S. (2012). Genome analysis of a simultaneously predatory and prey-independent, novel *Bdellovibrio bacteriovorus* from the River Tiber, supports in silico predictions of both ancient and recent lateral gene transfer from diverse bacteria. *BMC Genomics* 13:670. 10.1186/1471-2164-13-670 23181807 PMC3539863

[B79] Hobot J. A. Carlemalm E. Villiger W. Kellenberger E. (1984). Periplasmic gel: New concept resulting from the reinvestigation of bacterial cell envelope ultrastructure by new methods. *J. Bacteriol.* 160 143–152. 10.1128/jb.160.1.143-152.1984 6207168 PMC214693

[B80] Hoshino S. Onaka H. Abe I. (2019). Activation of silent biosynthetic pathways and discovery of novel secondary metabolites in actinomycetes by co-culture with mycolic acid-containing bacteria. *J. Ind. Microbiol. Biotechnol.* 46 363–374. 10.1007/s10295-018-2100-y 30488365

[B81] Hoshino S. Wakimoto T. Onaka H. Abe I. (2015). Chojalactones A–C, cytotoxic butanolides isolated from *Streptomyces* sp. Cultivated with mycolic acid containing bacterium. *Org. Lett.* 17 1501–1504. 10.1021/acs.orglett.5b00385 25742189

[B82] Ibrahimi M. (2020). *Extraction et caractérisation de nouveaux antibactériens produits par les actinobactéries prédatrices d’origine marine*. Doctoral dissertation, Marrakech: Université Cadi Ayyad.

[B83] Ibrahimi M. Korichi W. Hafidi M. Lemee L. Ouhdouch Y. Loqman S. (2020). Marine actinobacteria: Screening for predation leads to the discovery of potential new drugs against multidrug-resistant bacteria. *Antibiotics* 9:91. 10.3390/antibiotics9020091 32092889 PMC7168292

[B84] Ibrahimi M. Korichi W. Loqman S. Hafidi M. Ouhdouch Y. Lemee L. (2019). Thermochemolysis–GC-MS as a tool for chemotaxonomy and predation monitoring of a predatory Actinobacteria against a multidrug resistant bacteria. *J. Anal. Appl. Pyrolysis* 145:104740. 10.1016/j.jaap.2019.104740

[B85] Iebba V. Totino V. Santangelo F. Gagliardi A. Ciotoli L. Virga A. (2014). *Bdellovibrio bacteriovorus* directly attacks *Pseudomonas aeruginosa* and *Staphylococcus aureus Cystic fibrosis* isolates. *Front. Microbiol.* 5:280. 10.3389/fmicb.2014.00280 24926292 PMC4046265

[B86] Irschik H. Jansen R. Höfle G. Gerth K. Reichenbach H. (1985). The corallopyronins, new inhibitors of bacterial RNA synthesis from myxobacteria. *J. Antibiot.* 38 145–152. 10.7164/antibiotics.38.145 2581926

[B87] Jafarian N. Sepahi A. A. Naghavi N. S. Hosseini F. Nowroozi J. (2020). Using autochthonous *Bdellovibrio* as a predatory bacterium for reduction of Gram-negative pathogenic bacteria in urban wastewater and reuse it. *Iran. J. Microbiol.* 12:556. 10.18502/ijm.v12i6.5030 33613910 PMC7884277

[B88] Jamiołkowska A. (2020). Natural compounds as elicitors of plant resistance against diseases and new biocontrol strategies. *Agronomy* 10:173. 10.3390/agronomy10020173

[B89] Jia P. Zhou Y. Zhang X. Zhang Y. Dai R. (2018). Cyanobacterium removal and control of algal organic matter (AOM) release by UV/H2O2 pre-oxidation enhanced Fe (II) coagulation. *Water Res.* 131 122–130. 10.1016/j.watres.2017.12.020 29277080

[B90] Jousset A. (2012). Ecological and evolutive implications of bacterial defences against predators. *Environ. Microbiol.* 14 1830–1843. 10.1111/j.1462-2920.2011.02627.x 22040156

[B91] Jurkevitch E. (2007). Predatory behaviors in bacteria-diversity and transitions. *Microbe Am. Soc. Microbiol.* 2:67. 10.1128/microbe.2.67.1

[B92] Jurkevitch E. Davidov Y. (2006). “Phylogenetic diversity and evolution of predatory prokaryotes,” in *Predatory prokaryotes microbiology monographs*, ed. Jurkevitch E. (Berlin: Springer), 11–56. 10.1007/7171_052

[B93] Jurkevitch E. Ramati B. (2000). Design and uses of *Bdellovibrio* 16S rRNA-targeted oligonucleotides. *FEMS Microbiol. Lett.* 184 265–271. 10.1111/j.1574-6968.2000.tb09025.x 10713432

[B94] Jurkevitch E. Minz D. Ramati B. Barel G. (2000). Prey range characterization, ribotyping, and diversity of soil and rhizosphere *Bdellovibrio* spp. isolated on phytopathogenic bacteria. *Appl. Environ. Microbiol.* 66 2365–2371. 10.1128/AEM.66.6.2365-2371.2000 10831412 PMC110534

[B95] Kadouri D. E. To K. Shanks R. M. Doi Y. (2013). Predatory bacteria: A potential ally against multidrug-resistant Gram-negative pathogens. *PLoS One* 8:e63397. 10.1371/journal.pone.0063397 23650563 PMC3641118

[B96] Kadouri D. Venzon N. C. O’Toole G. A. (2007). Vulnerability of pathogenic biofilms to *Micavibrio aeruginosavorus*. *Appl. Environ. Microbiol.* 73 605–614. 10.1128/AEM.01893-06 17098913 PMC1796979

[B97] Katz L. Baltz R. H. (2016). Natural product discovery: Past, present, and future. *J. Ind. Microbiol. Biotechnol.* 43 155–176. 10.1007/s10295-015-1723-5 26739136

[B98] Keane R. Berleman J. (2016). The predatory life cycle of *Myxococcus xanthus*. *Microbiology* 162 1–11. 10.1099/mic.0.000208 26518442

[B99] Kessel M. Shilo M. (1976). Relationship of *Bdellovibrio* elongation and fission to host cell size. *J. Bacteriol.* 128 477–480. 10.1128/jb.128.1.477-480.1976 789349 PMC232876

[B100] Kiss H. Nett M. Domin N. Martin K. Maresca J. A. Copeland A. (2011). Complete genome sequence of the filamentous gliding predatory bacterium *Herpetosiphon aurantiacus* type strain (114-95 T). *Stand. Genomic Sci.* 5:356. 10.4056/sigs.2194987 22675585 PMC3368417

[B101] Kohl W. Irschik H. Reichenbach H. Höfle G. (1983). Antibiotika aus Gleitenden Bakterien, XVII. Myxopyronin A und B – zwei neue Antibiotika aus *Myxococcus fulvus* Stamm Mx f50. *Liebigs Ann. Chem.* 1983 1656–1667. 10.1002/jlac.198319831003

[B102] Korichi W. Ibrahimi M. Loqman S. Ouhdouch Y. Younes K. Lemée L. (2021). Assessment of Actinobacteria use in the elimination of multidrug-resistant bacteria of Ibn Tofail hospital wastewater (Marrakesh, Morocco): A chemometric data analysis approach. *Environ. Sci. Pollut. Res.* 28 26840–26848. 10.1007/s11356-021-12445-4 33501577

[B103] Korp J. Gurovic M. S. V. Nett M. (2016). Antibiotics from predatory bacteria. *Beilstein J. Organ. Chem.* 12:594. 10.3762/bjoc.12.58 27340451 PMC4902038

[B104] Koval S. F. Hynes S. H. Flannagan R. S. Pasternak Z. Davidov Y. Jurkevitch E. (2013). *Bdellovibrio exovorus* sp. nov., a novel predator of *Caulobacter crescentus*. *Int. J. Syst. Evol. Microbiol.* 63 146–151. 10.1099/ijs.0.039701-0 22368169

[B105] Kreutzer M. F. Kage H. Nett M. (2012). Structure and biosynthetic assembly of cupriachelin, a photoreactive siderophore from the bioplastic producer *Cupriavidus necator* H16. *J. Am. Chem. Soc.* 134 5415–5422. 10.1021/ja300620z 22381697

[B106] Krug D. Zurek G. Revermann O. Vos M. Velicer G. J. Müller R. (2008). Discovering the hidden secondary metabolome of *Myxococcus xanthus*: A study of intraspecific diversity. *Appl. Environ. Microbiol.* 74 3058–3068. 10.1128/AEM.02863-07 18378661 PMC2394937

[B107] Kumbhar C. Watve M. (2013). Why antibiotics: A comparative evaluation of different hypotheses for the natural role of antibiotics and an evolutionary synthesis. *Nat. Sci.* 5 26–40. 10.4236/ns.2013.54A005

[B108] Kumbhar C. Mudliar P. Bhatia L. Kshirsagar A. Watve M. (2014). Widespread predatory abilities in the genus *Streptomyces*. *Arch. Microbiol.* 196 235–248. 10.1007/s00203-014-0961-7 24535490

[B109] Kunze B. Bedorf N. Kohl W. Höfle G. Reichenbach H. (1989). Myxochelin A, a new iron-chelating compound from Angiococcus disciformis (Myxobacterales). Production, isolation, physico-chemical and biological properties. *J. Antibiot.* 42 14–17. 10.7164/antibiotics.42.14 2493439

[B110] Kunze B. Reichenbach H. Augustiniak H. Höfle G. (1982). Isolation and identification of althiomycin from *Cystobacter fuscus* (Myxobacterales). *J. Antibiot.* 35 635–636. 10.7164/antibiotics.35.635 6809724

[B111] Laloux G. (2020). Shedding light on the cell biology of the predatory bacterium *Bdellovibrio bacteriovorus*. *Front. Microbiol.* 10:3136. 10.3389/fmicb.2019.03136 32038570 PMC6985089

[B112] Lerner T. R. Lovering A. L. Bui N. K. Uchida K. Aizawa S.-I. Vollmer W. (2012). Specialized peptidoglycan hydrolases sculpt the intra-bacterial niche of predatory *Bdellovibrio* and increase population fitness. *PLoS Pathog.* 8:e1002524. 10.1371/journal.ppat.1002524 22346754 PMC3276566

[B113] Li S. Jochum C. C. Yu F. Zaleta-Rivera K. Du L. Harris S. D. (2008). An antibiotic complex from *Lysobacter enzymogenes* strain C3: Antimicrobial activity and role in plant disease control. *Phytopathology* 98 695–701. 10.1094/PHYTO-98-6-0695 18944294

[B114] Liu D. Yan R. Fu Y. Wang X. Zhang J. Xiang W. (2019). Antifungal, plant growth-promoting, and genomic properties of an endophytic actinobacterium *Streptomyces* sp. NEAU-S7GS2. *Front. Microbiol.* 10:2077. 10.3389/fmicb.2019.02077 31551997 PMC6746918

[B115] Machado J. Campos A. Vasconcelos V. Freitas M. (2017). Effects of microcystin-LR and cylindrospermopsin on plant-soil systems: A review of their relevance for agricultural plant quality and public health. *Environ. Res.* 153 191–204. 10.1016/j.envres.2016.09.015 27702441

[B116] Makkar N. S. Casida L. E. Jr. (1987). *Cupriavidus necator* gen. nov., sp. nov.; a nonobligate bacterial predator of bacteria in soil. *Int. J. Syst. Evol. Microbiol.* 37 323–326. 10.1099/00207713-37-4-323

[B117] Markelova N. Y. (2010). Predacious bacteria, *Bdellovibrio* with potential for biocontrol. *Int. J. Hygiene Environ. Health* 213 428–431. 10.1016/j.ijheh.2010.08.004 20850380

[B118] Marshall R. C. Whitworth D. E. (2019). Is “Wolf-Pack” predation by antimicrobial bacteria cooperative? Cell behaviour and predatory mechanisms indicate profound selfishness, even when working alongside kin. *Bioessays* 41:1800247. 10.1002/bies.201800247 30919490

[B119] Martin M. O. (2002). Predatory prokaryotes: An emerging research opportunity. *J. Mol. Microbiol. Biotechnol.* 4 467–478. 12432957

[B120] Martins S. J. Taerum S. J. Triplett L. Emerson J. B. Zasada I. de Toledo B. F. (2022). Predators of soil bacteria in plant and human health. *Phytobiomes J.* 6 184–200. 10.1094/PBIOMES-11-21-0073-RVW

[B121] Maurhofer M. Reimmann C. Schmidli-Sacherer P. Heeb S. Haas D. Défago G. (1998). Salicylic acid biosynthetic genes expressed in *Pseudomonas fluorescens* strain P3 improve the induction of systemic resistance in tobacco against tobacco necrosis virus. *Phytopathology* 88 678–684. 10.1094/PHYTO.1998.88.7.678 18944940

[B122] Mawang C. I. Azman A. S. Fuad A. S. M. Ahamad M. (2021). Actinobacteria: An eco-friendly and promising technology for the bioaugmentation of contaminants. *Biotechnol. Rep.* 32:e00679. 10.1016/j.btre.2021.e00679 34660214 PMC8503819

[B123] McBride M. J. Zusman D. R. (1996). Behavioral analysis of single cells of *Myxococcus xanthus* in response to prey cells of *Escherichia coli*. *FEMS Microbiol. Lett.* 137 227–231. 10.1111/j.1574-6968.1996.tb08110.x 8998990

[B124] McBride M. J. Xie G. Martens E. C. Lapidus A. Henrissat B. Rhodes R. G. (2009). Novel features of the polysaccharide-digesting gliding bacterium *Flavobacterium johnsoniae* as revealed by genome sequence analysis. *Appl. Environ. Microbiol.* 75 6864–6875. 10.1128/AEM.01495-09 19717629 PMC2772454

[B125] Meiser P. Bode H. B. Müller R. (2006). The unique DKxanthene secondary metabolite family from the myxobacterium *Myxococcus xanthus* is required for developmental sporulation. *Proc. Natl. Acad. Sci. U.S.A.* 103 19128–19133. 10.1073/pnas.0606039103 17148609 PMC1748187

[B126] Melaram R. Newton A. R. Chafin J. (2022). Microcystin contamination and toxicity: Implications for agriculture and public health. *Toxins* 14:350. 10.3390/toxins14050350 35622596 PMC9145844

[B127] Mendes-Soares H. Velicer G. J. (2013). Decomposing predation: Testing for parameters that correlate with predatory performance by a social bacterium. *Microbial Ecol.* 65 415–423. 10.1007/s00248-012-0135-6 23184156 PMC3563865

[B128] Miyanaga S. Obata T. Onaka H. Fujita T. Saito N. Sakurai H. (2006). Absolute configuration and antitumor activity of myxochelin a produced by *Nonomuraea pusilla* TP-A0861†. *J. Antibiot.* 59 698–703. 10.1038/ja.2006.93 17256468

[B129] Monnappa A. K. Dwidar M. Seo J. K. Hur J.-H. Mitchell R. J. (2014). *Bdellovibrio bacteriovorus* inhibits *Staphylococcus aureus* biofilm formation and invasion into human epithelial cells. *Sci. Rep.* 4:3811. 10.1038/srep03811 24448451 PMC3898049

[B130] Mookherjee A. Jurkevitch E. (2022). Interactions between *Bdellovibrio* and like organisms and bacteria in biofilms: Beyond predator–prey dynamics. *Environ. Microbiol.* 24 998–1011. 10.1111/1462-2920.15844 34816563

[B131] Moreira C. Ramos V. Azevedo J. Vasconcelos V. (2014). Methods to detect cyanobacteria and their toxins in the environment. *Appl. Microbiol. Biotechnol.* 98 8073–8082. 10.1007/s00253-014-5951-9 25085613

[B132] Moulder J. W. (1985). Comparative biology of intracellular parasitism. *Microbiol. Rev.* 49:298. 10.1128/mr.49.3.298-337.1985 3900672 PMC373037

[B133] Nair R. (2016). *Biotic interactions of Myxococcus xanthus*. Ph.D. dissertation. Zurich: ETH Zürich.

[B134] Nakano C. Oshima M. Kurashima N. Hoshino T. (2015). Identification of a new diterpene biosynthetic gene cluster that produces O-Methylkolavelool in *Herpetosiphon aurantiacus*. *ChemBioChem* 16 772–781. 10.1002/cbic.201402652 25694050

[B135] Nett M. Erol Ö Kehraus S. Köck M. Krick A. Eguereva E. (2006). Siphonazole, an unusual metabolite from *Herpetosiphon* sp. *Angew. Chemie Int. Ed.* 45 3863–3867. 10.1002/anie.200504525 16671154

[B136] O’Neil J. M. Davis T. W. Burford M. A. Gobler C. J. (2012). The rise of harmful cyanobacteria blooms: The potential roles of eutrophication and climate change. *Harmful Algae* 14 313–334. 10.1016/j.hal.2011.10.027

[B137] O’Sullivan J. McCullough J. E. Tymiak A. A. Kirsch D. R. Trejo W. H. Principe P. A. (1988). Lysobactin, a novel antibacterial agent produced by *Lysobacter* sp. *J. Antibiot.* 41 1740–1744. 10.7164/antibiotics.41.1740 3209465

[B138] Octaviana S. (2021). *Exploring the diversity and antimicrobial potential of predatory bacteria from Indonesian mangroves*. Ph.D. dissertation. Braunschweig: Universitätsbibliothek Braunschweig.

[B139] Ogundero A. Vignola M. Connelly S. Sloan W. T. (2022). Validating flow cytometry as a method for quantifying *Bdellovibrio* predatory bacteria and its prey for microbial ecology. *Microbiol. Spectrum* 10:e01033-21. 10.1128/spectrum.01033-21 35196816 PMC8865432

[B140] Olanya O. M. Lakshman D. K. (2015). Potential of predatory bacteria as biocontrol agents for foodborne and plant pathogens. *J. Plant Pathol.* 97 405–417.

[B141] Olsen L. Choffnes E. R. Relman D. A. Pray L. (2011). *Fungal diseases: An emerging threat to human, animal and plant health*. Workshop summary. Washington, DC: National Academies Press. 22259817

[B142] Ongena M. Jacques P. (2008). *Bacillus lipopeptides*: Versatile weapons for plant disease biocontrol. *Trends Microbiol.* 16 115–125. 10.1016/j.tim.2007.12.009 18289856

[B143] Ortiz-Ortiz L. Bojalil L. F. Yakoleff V. (2013). *Biological, biochemical, and biomedical aspects of actinomycetes.* Amsterdam: Elsevier.

[B144] Ouchari L. Boukeskasse A. Bouizgarne B. Ouhdouch Y. (2019). Antimicrobial potential of actinomycetes isolated from the unexplored hot Merzouga desert and their taxonomic diversity. *Biol. Open* 8:bio035410. 10.1242/bio.035410 30127092 PMC6398458

[B145] Palaniyandi S. A. Yang S. H. Zhang L. Suh J. W. (2013). Effects of Actinobacteria on plant disease suppression and growth promotion. *Appl. Microbiol. Biotechnol.* 97 9621–9636. 10.1007/s00253-013-5206-1 24092003

[B146] Pandit M. A. Kumar J. Gulati S. Bhandari N. Mehta P. Katyal R. (2022). Major biological control strategies for plant pathogens. *Pathogens* 11:273. 10.3390/pathogens11020273 35215215 PMC8879208

[B147] Parry J. D. (2004). Protozoan grazing of freshwater biofilms. *Adv. Appl. Microbiol.* 54 167–196. 10.1016/S0065-2164(04)54007-8 15251281

[B148] Pasternak Z. Njagi M. Shani Y. Chanyi R. Rotem O. Lurie-Weinberger M. N. (2014). In and out: An analysis of epibiotic vs periplasmic bacterial predators. *ISME J.* 8 625–635. 10.1038/ismej.2013.164 24088628 PMC3930308

[B149] Pérez J. Contreras-Moreno F. J. Marcos-Torres F. J. Moraleda-Muñoz A. Muñoz-Dorado J. (2020). The antibiotic crisis: How bacterial predators can help. *Comput. Struct. Biotechnol. J.* 18 2547–2555. 10.1016/j.csbj.2020.09.010 33033577 PMC7522538

[B150] Pérez J. Moraleda-Muñoz A. Marcos-Torres F. J. Muñoz-Dorado J. (2016). Bacterial predation: 75 years and counting! *Environ. Microbiol.* 18 766–779. 10.1111/1462-2920.13171 26663201

[B151] Plaza A. Viehrig K. Garcia R. Müller R. (2013). Jahnellamides, α-Keto-β-methionine-containing peptides from the terrestrial myxobacterium *Jahnella* sp.: Structure and biosynthesis. *Org. Lett.* 15 5882–5885. 10.1021/ol402967y 24199909

[B152] Quinn G. R. Skerman V. B. (1980). Herpetosiphon—nature’s scavenger? *Curr. Microbiol.* 4 57–62. 10.1007/BF02602893

[B153] Rani L. Thapa K. Kanojia N. Sharma N. Singh S. Grewal A. S. (2021). An extensive review on the consequences of chemical pesticides on human health and environment. *J. Clean. Prod.* 283:124657. 10.1016/j.jclepro.2020.124657

[B154] Rashidan K. K. Bird D. F. (2001). Role of predatory bacteria in the termination of a cyanobacterial bloom. *Microb. Ecol.* 41 97–105. 10.1007/s002480000074 12032614

[B155] Raza W. Yang W. Shen Q. R. (2008). *Paenibacillus polymyxa*: Antibiotics, hydrolytic enzymes and hazard assessment. *J. Plant Pathol.* 90 419–430.

[B156] Reichenbach H. Höfle G. (1993). Biologically active secondary metabolites from myxobacteria. *Biotechnol. Adv.* 11 219–277. 10.1016/0734-9750(93)90042-L 14545007

[B157] Richards G. P. Fay J. P. Dickens K. A. Parent M. A. Soroka D. S. Boyd E. F. (2012). Predatory bacteria as natural modulators of *Vibrio parahaemolyticus* and *Vibrio vulnificus* in seawater and oysters. *Appl. Environ. Microbiol.* 78 7455–7466. 10.1128/AEM.01594-12 22904049 PMC3457099

[B158] Richards G. P. Watson M. A. Boyd E. F. Burkhardt W. Lau R. Uknalis J. (2013). Seasonal levels of the *Vibrio* predator *Bacteriovorax* in Atlantic, Pacific, and gulf coast seawater. *Int. J. Microbiol.* 2013:375371. 10.1155/2013/375371 24454382 PMC3881529

[B159] Ripple W. J. Beschta R. L. (2004). Wolves and the ecology of fear: Can predation risk structure ecosystems? *BioScience* 54 755–766. 10.1641/0006-3568(2004)054[0755:WATEOF]2.0.CO;2 33389153

[B160] Rosenberg E. Varon M. (1984). “Antibiotics and lytic enzymes,” in *Myxobacteria*, ed. Rosenberg E. (New York, NY: Springer), 109–125. 10.1007/978-1-4613-8280-5_5

[B161] Ryan R. P. Monchy S. Cardinale M. Taghavi S. Crossman L. Avison M. B. (2009). The versatility and adaptation of bacteria from the genus *Stenotrophomonas*. *Nat. Rev. Microbiol.* 7 514–525. 10.1038/nrmicro2163 19528958

[B162] Santos-Aberturas J. Vior N. M. (2022). Beyond soil-dwelling actinobacteria: Fantastic antibiotics and where to find them. *Antibiotics* 11:195. 10.3390/antibiotics11020195 35203798 PMC8868522

[B163] Saraf S. R. Frenkel A. Harke M. J. Jankowiak J. G. Gobler C. J. McElroy A. E. (2018). Effects of microcystis on development of early life stage Japanese medaka (*Oryzias latipes*): Comparative toxicity of natural blooms, cultured Microcystis and microcystin-LR. *Aquat. Toxicol.* 194 18–26. 10.1016/j.aquatox.2017.10.026 29132031

[B164] Sathya A. Vijayabharathi R. Gopalakrishnan S. (2017). Plant growth-promoting Actinobacteria: A new strategy for enhancing sustainable production and protection of grain legumes. *3 Biotech* 7 1–10. 10.1007/s13205-017-0736-3 28560641 PMC5449283

[B165] Saw J. H. Yuryev A. Kanbe M. Hou S. Young A. G. Aizawa S.-I. (2012). Complete genome sequencing and analysis of *Saprospira grandis* str. Lewin, a predatory marine bacterium. *Stand. Genomic Sci.* 6:84. 22675601 10.4056/sigs.2445005 PMC3368406

[B166] Saxon E. B. Jackson R. W. Bhumbra S. Smith T. Sockett R. E. (2014). *Bdellovibrio bacteriovorus* HD100 guards against *Pseudomonas* tolaasii brown-blotch lesions on the surface of post-harvest *Agaricus bisporus* supermarket mushrooms. *BMC Microbiol.* 14:163. 10.1186/1471-2180-14-163 24946855 PMC4077555

[B167] Schäberle T. F. Schmitz A. Zocher G. Schiefer A. Kehraus S. Neu E. (2015). Insights into structure–activity relationships of bacterial RNA polymerase inhibiting corallopyronin derivatives. *J. Nat. Prod.* 78 2505–2509. 10.1021/acs.jnatprod.5b00175 26431157

[B168] Scherff R. H. (1973). Control of bacterial blight of soybean by *Bdellovibrio bacteriovorus*. *Phytopathology* 63 400–402. 10.1094/Phyto-63-400

[B169] Scherlach K. Hertweck C. (2009). Triggering cryptic natural product biosynthesis in microorganisms. *Org. Biomol. Chem.* 7 1753–1760. 10.1039/b821578b 19590766

[B170] Schieferdecker S. Domin N. Hoffmeier C. Bryant D. A. Roth M. Nett M. (2015). Structure and absolute configuration of Auriculamide, a natural product from the predatory bacterium *Herpetosiphon aurantiacus*. *Eur. J. Org. Chem.* 2015 3057–3062. 10.1002/ejoc.201500181

[B171] Schieferdecker S. König S. Weigel C. Dahse H.-M. Werz O. Nett M. (2014). Structure and biosynthetic assembly of gulmirecins, macrolide antibiotics from the predatory bacterium *Pyxidicoccus fallax*. *Chem. Eur. J.* 20 15933–15940. 10.1002/chem.201404291 25287056

[B172] Schisler D. A. Khan N. I. Boehm M. J. Slininger P. J. (2002). Greenhouse and field evaluation of biological control of *Fusarium* head blight on durum wheat. *Plant Dis.* 86 1350–1356. 10.1094/PDIS.2002.86.12.1350 30818440

[B173] Schwudke D. Strauch E. Krueger M. Appel B. (2001). Taxonomic studies of predatory *Bdellovibrio*s based on 16S rRNA analysis, ribotyping and the hit locus and characterization of isolates from the gut of animals. *Syst. Appl. Microbiol.* 24 385–394. 10.1078/0723-2020-00042 11822674

[B174] Seccareccia I. Kost C. Nett M. (2015). Quantitative analysis of *Lysobacter* predation. *Appl. Environ. Microbiol.* 81 7098–7105. 10.1128/AEM.01781-15 26231654 PMC4579460

[B175] Shi W. Köhler T. Zusman D. R. (1993). Chemotaxis plays a role in the social behaviour of *Myxococcus xanthus*. *Mol. Microbiol.* 9 601–611. 10.1111/j.1365-2958.1993.tb01720.x 8412706

[B176] Shilo M. (1967). Formation and mode of action of algal toxins. *Bacteriol. Rev.* 31 180–193. 10.1128/br.31.3.180-193.1967 4864729 PMC378282

[B177] Shin D. Byun W. S. Moon K. Kwon Y. Bae M. Um S. (2018). Coculture of marine *Streptomyces* sp. with *Bacillus* sp. Produces a new piperazic acid-bearing cyclic peptide. *Front. Chem.* 6:498. 10.3389/fchem.2018.00498 30406080 PMC6201156

[B178] Shivlata L. Satyanarayana T. (2017). “Actinobacteria in agricultural and environmental sustainability,” in *Agro-environmental sustainability*, eds Singh J. Seneviratne G. (Cham: Springer), 173–218. 10.1007/978-3-319-49724-2_9

[B179] Sinclair A. R. E. Mduma S. Brashares J. S. (2003). Patterns of predation in a diverse predator–prey system. *Nature* 425 288–290. 10.1038/nature01934 13679915

[B180] Sockett R. E. (2009). Predatory lifestyle of *Bdellovibrio bacteriovorus*. *Annu. Rev. Microbiol.* 63 523–539. 10.1146/annurev.micro.091208.073346 19575566

[B181] Sockett R. E. Lambert C. (2004). *Bdellovibrio* as therapeutic agents: A predatory renaissance? *Nat. Rev. Microbiol.* 2 669–675. 10.1038/nrmicro959 15263901

[B182] Stolp H. Starr M. P. (1963). *Bdellovibrio bacteriovorus* gen. et sp. n., a predatory, ectoparasitic, and bacteriolytic microorganism. *Antonie Van Leeuwenhoek* 29 217–248. 10.1007/BF02046064 14068454

[B183] Sudo S. Dworkin M. (1972). Bacteriolytic enzymes produced by *Myxococcus xanthus*. *J. Bacteriol.* 110 236–245. 10.1128/jb.110.1.236-245.1972 4622898 PMC247403

[B184] Sun R. Sun P. Zhang J. Esquivel-Elizondo S. Wu Y. (2018). Microorganisms-based methods for harmful algal blooms control: A review. *Bioresour. Technol.* 248 12–20. 10.1016/j.biortech.2017.07.175 28801171

[B185] Sung A. Gromek S. Balunas M. (2017). Upregulation and identification of antibiotic activity of a marine-derived *Streptomyces* sp. via co-cultures with human pathogens. *Mar. Drugs* 15:250. 10.3390/md15080250 28800088 PMC5577605

[B186] Swain D. M. Yadav S. K. Tyagi I. Kumar R. Kumar R. Ghosh S. (2017). A prophage tail-like protein is deployed by *Burkholderia* bacteria to feed on fungi. *Nat. Commun.* 8 1–9. 10.1038/s41467-017-00529-0 28864820 PMC5581363

[B187] Thiery S. Kaimer C. (2020). The predation strategy of *Myxococcus xanthus*. *Front. Microbiol.* 11:2. 10.3389/fmicb.2020.00002 32010119 PMC6971385

[B188] Thomashow M. F. Rittenberg S. C. (1978). Penicillin-induced formation of osmotically stable spheroplasts in nongrowing *Bdellovibrio bacteriovorus*. *J. Bacteriol.* 133 1484–1491. 10.1128/jb.133.3.1484-1491.1978 641013 PMC222189

[B189] Trick I. Lingens F. (1984). Characterization of *Herpetosiphon* spec. —A gliding filamentous bacterium from bulking sludge. *Appl. Microbiol. Biotechnol.* 19 191–198. 10.1007/BF00256453

[B190] Verheecke C. Liboz T. Anson P. Zhu Y. Mathieu F. (2015). *Streptomyces*–*Aspergillus flavus* interactions: Impact on aflatoxin B accumulation. *Food Addit. Contam. Part A* 32 572–576. 10.1080/19440049.2014.1003336 25632796

[B191] Vikeli E. Widdick D. A. Batey S. F. Heine D. Holmes N. A. Bibb M. J. (2019). In situ activation and heterologous production of a cryptic lantibiotic from a plant-ant derived *Saccharopolyspora* species. *Appl. Environ. Microbiol.* 86:e01876-19. 10.1128/AEM.01876-19 31732571 PMC6974636

[B192] Wakefield J. Hassan H. M. Jaspars M. Ebel R. Rateb M. E. (2017). Dual induction of new microbial secondary metabolites by fungal bacterial co-cultivation. *Front. Microbiol.* 8:1284. 10.3389/fmicb.2017.01284 28744271 PMC5504103

[B193] Waksman S. A. Woodruff H. B. (1941). Actinomyces antibioticus, a new soil organism antagonistic to pathogenic and non-pathogenic bacteria. *J. Bacteriol.* 42:231. 10.1128/jb.42.2.231-249.1941 16560451 PMC374755

[B194] Wenzel S. C. Müller R. (2009). Myxobacteria—‘microbial factories’ for the production of bioactive secondary metabolites. *Mol. BioSyst.* 5 567–574. 10.1039/b901287g 19462013

[B195] Whitworth D. E. (2022). Myxobacteria: Physiology and regulation. *Microorganisms* 10:805. 10.3390/microorganisms10040805 35456855 PMC9032285

[B196] Wu C. Zacchetti B. Ram A. F. Van Wezel G. P. Claessen D. Hae Choi Y. (2015). Expanding the chemical space for natural products by *Aspergillus*-*Streptomyces* co-cultivation and biotransformation. *Sci. Rep.* 5 1–10. 10.1038/srep10868 26040782 PMC4455117

[B197] Wu Z. Li Y. Chen H. Rao J. Sun Q. (2022). Effects of straw mulching on predatory myxobacterial communities in different soil aggregates under wheat-corn rotation. *Environ. Sci. Pollut. Res.* 29 29062–29074. 10.1007/s11356-021-18350-0 34993829

[B198] Xiao Y. Gerth K. Müller R. Wall D. (2012). Myxobacterium-produced antibiotic TA (myxovirescin) inhibits type II signal peptidase. *Antimicrob. Agents Chemother.* 56 2014–2021. 10.1128/AAC.06148-11 22232277 PMC3318312

[B199] Xiao Y. Wei X. Ebright R. Wall D. (2011). Antibiotic production by myxobacteria plays a role in predation. *J. Bacteriol.* 193 4626–4633. 10.1128/JB.05052-11 21764930 PMC3165673

[B200] Yakimov M. M. Merkel A. Y. Gaisin V. A. Pilhofer M. Messina E. Hallsworth J. E. (2022). Cultivation of a vampire: ‘*Candidatus* Absconditicoccus praedator’. *Environ. Microbiol.* 24 30–49. 10.1111/1462-2920.15823 34750952

[B201] Ye X. Li Z. Luo X. Wang W. Li Y. Li R. (2020). A predatory myxobacterium controls cucumber *Fusarium* wilt by regulating the soil microbial community. *Microbiome* 8 1–17. 10.1186/s40168-020-00824-x 32252828 PMC7137222

[B202] Yu M. Li Y. Banakar S. P. Liu L. Shao C. Li Z. (2019). New metabolites from the co-culture of marine-derived actinomycete *Streptomyces rochei* MB037 and fungus *Rhinocladiella similis* 35. *Front. Microbiol.* 10:915. 10.3389/fmicb.2019.00915 31134000 PMC6514141

[B203] Yu Y. Zeng Y. Li J. Yang C. Zhang X. Luo F. (2019). An algicidal *Streptomyces amritsarensis* strain against *Microcystis aeruginosa* strongly inhibits microcystin synthesis simultaneously. *Sci. Total Environ.* 650 34–43. 10.1016/j.scitotenv.2018.08.433 30195130

[B204] Zeng Y. Wang J. Yang C. Ding M. Hamilton P. B. Zhang X. (2021). A *Streptomyces globisporus* strain kills *Microcystis aeruginosa* via cell-to-cell contact. *Sci. Total Environ.* 769:144489. 10.1016/j.scitotenv.2020.144489 33465632

[B205] Zeph L. R. Casida L. E. (1986). Gram-negative versus gram-positive (actinomycete) nonobligate bacterial predators of bacteria in soil. *Appl. Environ. Microbiol.* 52 819–823. 10.1128/aem.52.4.819-823.1986 16347175 PMC239120

